# Differential ErbB receptor dimerization modulates the ability of EGF receptor ligands to regulate metabolic flux

**DOI:** 10.1016/j.jbc.2026.113230

**Published:** 2026-06-04

**Authors:** Jennifer Macdonald-Obermann, Kevin Cho, Gary J. Patti, Linda J. Pike

**Affiliations:** 1 Department of Biochemistry and Molecular Biophysics, Washington University School of Medicine, St Louis, Missouri, USA; 2 Department of Chemistry, Medicine, Center for Mass Spectrometry and Metabolic Tracing, Washington University, St Louis, Missouri, USA

**Keywords:** EGF receptor, tyrosine kinase, growth factor, hormone receptor, cell signaling, metabolomics

## Abstract

The epidermal growth factor (EGF) receptor tyrosine kinase binds seven different agonist ligands. It stimulates proliferation of MCF-7 cells while epigen and EPR, stimulate differentiation. This distinction is thought to be due to the phenomenon of ligand bias in which two agonists bind to the same receptor but generate different responses. These reported differences among EGFR ligands led us to examine their effects plus those of neureglin 2ß (NRG-2ß), an ErbB3-ErbB4 ligand, on metabolism in MCF-7 cells and MDA-MB-468 cells. We followed the flux of carbons from either [1,2-^13^C]-glucose or [U-^13^C]-glutamine through the early pathways of intermediary metabolism. EGF stimulated flux through glycolysis, the pentose phosphate pathway (PPP), and the tricarboxylic acid cycle in both lines. However, MCF-7 cells channeled ribose from the PPP into nucleotide biosynthesis whereas MDA-MB-468 cells recycled the riboses through the nonoxidative pathway of the PPP back into glycolysis. In MDA-MB-468 cells, all EGF receptor ligands induced a similar level of metabolic flux while NRG-2ß was inactive. By contrast, in MCF-7 cells, NRG-2ß, betacellulin, EPR, and epigen were significantly more effective at stimulating metabolic flux than the other EGF receptor ligands. Thus, bias was apparent in MCF-7 cells but not in MD-MB-468 cells. As the two lines express different complements of ErbB family receptors, we speculate that the differences in response are the result of different ligands preferentially inducing specific homodimer or heterodimer pairings. Our findings highlight the need to consider the possibility of system bias in cells when interpreting data related to ligand bias.

The epidermal growth factor (EGF) receptor is a classical receptor tyrosine kinase comprised of an extracellular ligand-binding domain and an intracellular tyrosine kinase domain (1). The receptor dimerizes upon binding an agonist ligand (2, 3, 4), resulting in the activation of the kinase domain (5). This leads to autophosphorylation of the receptor (6, 7, 8), generating binding sites for SH2 and PTB domain-containing proteins, which mediate the downstream effects of the agonist (9, 10, 11). In addition to promoting dimerization of the EGF receptor, agonist ligands also appear to be capable of inducing higher order oligomerization of the receptor (12, 13, 14). Such oligomerization may be important for the phosphorylation of some sites of autophosphorylation and the induction of some downstream signals (15).

The EGF receptor is a member of the ErbB family of receptors that also includes the homologous ErbB2, ErbB3, and ErbB4 receptors (reviewed in (16)). ErbB3 and ErbB4 bind several different agonist ligands; however, ErbB2 has no known ligand. Although ErbB3 can bind ligands, it has no intrinsic tyrosine kinase activity. Thus, for both ErbB2 and ErbB3, ligand-stimulated tyrosine kinase activation and subsequent signal transduction only occurs in the context of heterodimerization with another ErbB family member. Although essentially all heterodimers can be formed, multiple studies have suggested that ErbB2 is the preferred heterodimerization partner (17, 18). However, homodimerization of the EGF receptor or ErbB4 appears to be preferred over heterodimerization at least in some cases (19).

The EGF receptor binds seven different ligands (20, 21). This includes the high affinity ligands, EGF, transforming growth factor alpha (TGFα), heparin-binding EGF (HB-EGF), and betacellulin (BTC) as well as the low affinity ligands, amphiregulin (AREG), epiregulin (EPR), and epigen (EPG). Although all of the ligands bind to the same site on the EGF receptor (2, 3, 22), different ligands can induce different biological responses in the same cell. For example, in mammary epithelial cells, EGF induced development into both the luminal and myoepithelial lineages. However, AREG induced differentiation only toward the luminal lineage and TGFα resulted in differentiation only toward the myoepithelial lineage (23). In 32D/EGFR myeloid and MCF10A breast cells, TGFα, AREG, and EPG stimulated greater cell proliferation and DNA synthesis than did EGF, BTC, HB-EGF, and EPR (24). In human keratinocytes, TGFα, but not EGF, induced exosome-mediated secretion (25). And in MCF-7 cells, EGF induced cellular proliferation while EPG and EPR induced cellular differentiation (22). These observations have led to the concept of ligand bias in which one ligand biases the response of a cell toward one set of pathways leading to a particular biological outcome while a different ligand for the same receptor selectively activates a different set of downstream pathways leading to a different biological response.

Central to the ability of growth factors to promote cell proliferation and/or differentiation is their ability to regulate the metabolic pathways that produce energy and biomass for cell growth and survival. Little is known about how EGF receptor ligands regulate intermediary metabolism. In this study, we use metabolic flux experiments to examine how EGF alters glucose metabolism in MCF-7 and MDA-MB-468 cells, two cell lines widely used to study estrogen receptor-positive breast carcinoma or triple-negative breast cancer, respectively. We compared the effects of all EGF receptor agonists plus the ErbB3-ErbB4 ligand, NRG-2ß, on glucose metabolism to determine whether the agonist-specific differences in biological outcomes were preceded by differences in the ability of agonists to regulate metabolic flux. Our results reveal differences in the effects on metabolism of the seven EGF receptor-binding ligands in the two different breast cancer cell lines. MCF-7 cells exhibited characteristics normally ascribed to ligand bias; however, MDA-MB-468 cells showed no such ligand bias. The difference in ligand responsiveness in two different systems implies the presence of cell-specific features that contribute to the observed bias in the response to EGF receptor ligands. As the two lines express different complements of ErbB family receptors, we suggest that variations in the ability of agonists to promote the formation of specific receptor homo- or hetero-dimers make a major contribution to bias seen among EGF receptor ligands.

## Results

### ErbB receptors in breast cancer lines

MCF-7 cells are the most widely used model cell line for the study of estrogen-receptor positive breast cancer. They are classified as Luminal A breast carcinoma cells and exhibit features of the mammary epithelium (26). MDA-MB-468 cells are a line of triple-negative breast cancer cells classified as basal breast carcinoma cells (26). The triple-negative designation indicates that the cells lack estrogen receptors, progesterone receptors and do not exhibit amplification of ErbB2 (HER2).

**Figure 1:**
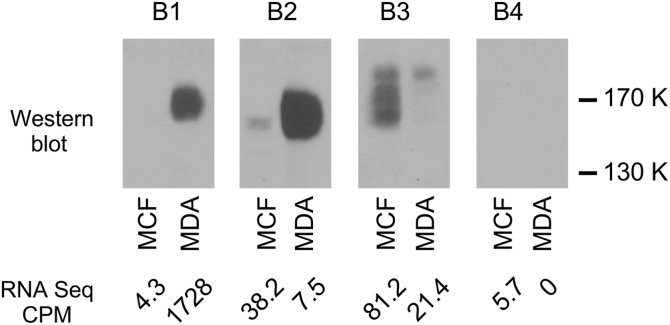
**ErbB receptor expression in MCF-7 cells and MDA-MB-468 cells.** Equal amounts of protein from MCF-7 and MDA-MB-468 cells were analyzed by SDS polyacrylamide gel electrophoresis and blotted for EGF receptor (B1), ErbB2 (B2), ErbB3 (B3), or ErbB4. RNA-seq analysis was performed as described in Experimental Procedures and is reported in counts per million by the numbers below each blot. For EGFR, ErbB2, and ErbB4 blots, 60 μg protein was loaded per well. For ErbB3 blot, 15 μg protein was loaded per well. EGF, epidermal growth factor; EGFR, epidermal growth factor receptor.

We first examined the level of the four ErbB family receptors in these cells using both Western blotting and RNA-Seq (Fig. 1). RNA-Seq was performed because of concerns over limits of detection and possible cross reactivity of ErbB receptor antibodies in Western blotting experiments. EGF receptor expression was readily detectable in MDA-MB-468 cells by Western blot but was undetectable in MCF-7 cells. We used ^125^I-EGF binding to determine that MCF-7 cells express ∼20,000 EGF receptors per cell *versus* ∼1 × 10^6^ receptors per cell in MDA-MB-468 cells. These numbers are in line with the data from RNA-Seq which indicate that MDA-MB-468 cells harbor several orders of magnitude more EGF receptor transcripts than do MCF-7 cells.

Western blotting showed low levels of ErbB2 expression in MCF-7 cells but unexpectedly high levels in MDA-MB-468 cells. As MDA-MB-468 cells are triple-negative breast cancer cells and thus do not have amplification of ErbB2, this suggests cross reactivity of the ErbB2 antibody with another ErbB family member, most likely the overexpressed EGF receptor in these cells. Consistent with the likelihood of antibody cross-reactivity and the triple-negative classification of MDA-MB-468 cells, RNA-Seq analysis showed low numbers of ErbB2 transcripts in MDA-MB-468, approximately 5-fold less than in MCF-7 cells.

Western blots for ErbB3 suggested higher levels of protein expression in MCF-7 cells than in MDA-MB-468 cells, consistent with the higher number of transcripts for ErbB3 detected in MCF-7 cells. ErbB4 levels were undetectable by Western blot in both cell lines. RNA-Seq demonstrated the presence of low levels of ErbB4 transcripts in MCF-7 cells but none in MDA-MB-468 cells. The clear discrepancy between Western blotting results and RNA-Seq results underscores the need for caution when determining ErbB receptor expression levels by Western blot.

### The effect of EGF on metabolism in MCF-7 cells

**Figure 2:**
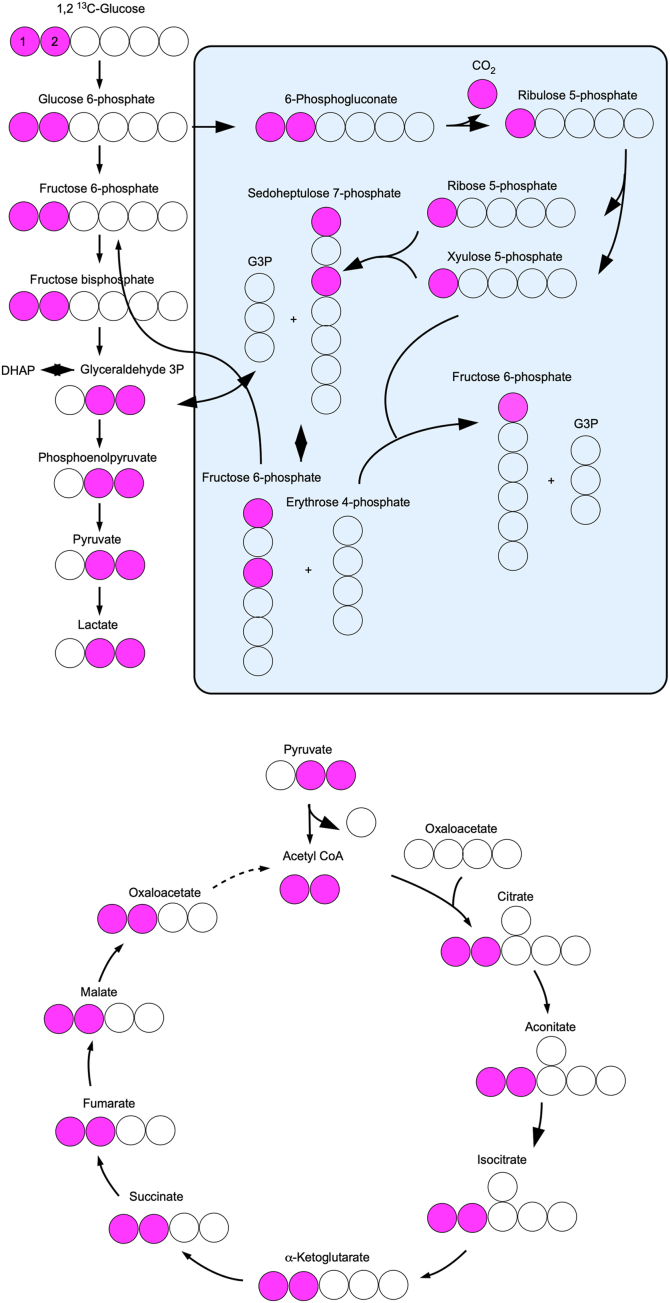
**Flow of carbons 1 and 2 from [1,2-^13^C]-glucose through glycolysis, the pentose phosphate pathway, and the TCA cycle.**^13^C-labeled carbons are shown as *magenta circles*. ^12^C-carbons are represented as *open circles*. (*upper panel*) Reactions of glycolysis are shown vertically to the left of the *blue box*. Reactions of the pentose phosphate pathway are shown within the *blue box*. m + 1 ribose-5P is used in the synthesis of ribonucleotides giving rise to the m + 1 isotopologues of all four ribonucleotides. (*lower panel*) Reactions of the TCA cycle are shown. The m + 2 pyruvate is generated from the metabolism of [1,2-^13^C]-glucose *via* glycolysis. The maximum enrichment of m + 2 pyruvate is 0.5 as only half of the dihydroxyacetone-P/glyceraldehyde-3P metabolites contain labeled carbons. Only the first cycle through the TCA cycle is shown. Additional isotopologues are generated in subsequent cycles as the m + 2 oxaloacetate combines with labeled or unlabeled acetyl-CoA. TCA, tricarboxylic acid.

To assess metabolic flux in MCF-7 cells, we first incubated cells in the presence of 5 mM [1,2-^13^C]-glucose for increasing lengths of time up to 4 h in the absence of any growth factor. Figure 2 traces the flow of the two labeled carbons through glycolysis, the pentose phosphate pathway (PPP), and the tricarboxylic acid (TCA) cycle. Fig. S1 shows the complete time course of the major isotopologue of a primary intermediate in glycolysis, the TCA cycle and the PPP to document the rate at which steady state labeling was achieved in each pathway. We also looked at flux into ribonucleotide pools which is due to the incorporation of labeled ribose-5P into ribonucleotides. As shown in Fig. S1, flux into the glycolytic and PPPs reached steady state by 20 min. Flux of label into the TCA was slower, requiring 2 to 4 h to achieve steady state while flux into nucleotide pools was slow, and was still rising at 4 h. We chose to use a 4-h labeling period for our experiments as most of the key pathways had reached steady state labeling under control conditions by that time. This minimizes any short-term effect on the uptake of label on longer-term effects of the growth factor on metabolism.

**Figure 3:**
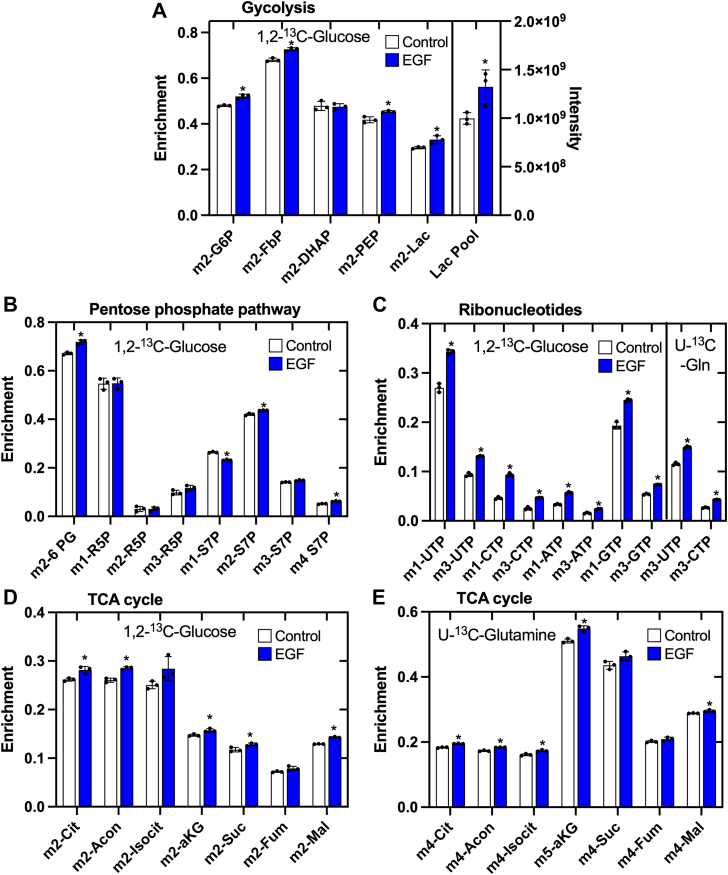
**EGF-stimulated flux of ^13^C-labeled glucose or glutamine through glycolysis, the pentose phosphate pathway, the TCA cycle, and ribonucleotides in MCF-7 cells.** MCF-7 cells were labeled in triplicate with [1,2-^13^C] glucose (panels *A* through *D*) or [U-^13^C] glutamine (panel *E*), stimulated with 30 nM EGF, and extracted for metabolomics analysis as outlined in Experimental procedures. *A*, glycolytic intermediates; *B*, pentose phosphate pathway intermediates; *C*, ribonucleotide triphosphates; *D*, TCA cycle intermediates in cells labeled with [1,2-^13^C] glucose; *E*, TCA cycle intermediates in cells labeled with [U-^13^C] glutamine. ∗ indicates that the difference between control and the EGF-treated sample was significant at the *p* < 0.05 level using unpaired, 2-tailed t-tests. Data points represent triplicate technical replicates from a single biological experiment that was repeated three times. EGF, epidermal growth factor; TCA, tricarboxylic acid.

Figure 3*A* shows the effects of EGF on the flux of label into the major isotopologues of glycolytic intermediates after a 4-h incubation in the absence or presence of EGF. EGF modestly but significantly stimulated flux into most of these intermediates. EGF also stimulated an increase in the pool of lactate, confirming that EGF increases the flux through glycolysis in MCF-7 cells. Although the absolute differences were modest for most of the metabolites studied here (ranging from a 4% to a 33% increase over untreated controls), even a small difference in flux will, over time, lead to a large difference in metabolite levels.

Flux of label from [1,2-^13^C]-glucose into a number of intermediates in the PPP (Fig. 3*B*) was also significantly stimulated by EGF. However, there was no significant increase of flux into any of the isotopologues of ribose-5P. As can be seen from the data in Figure 3*B*, the m + 1 isotopologue of ribose-5P was the major species of this metabolite and there was little flux into the m + 2 or m + 3 isotopologues. This suggests that there was relatively little use of the nonoxidative reactions of the PPP. Consistent with this interpretation, EGF actually reduced the flux of label into the m + 1 isotopologue of sedoheptulose-7P and had a minimal effect on flux into the m + 2, m + 3 or m + 4 isotopologues of this metabolite. However, EGF strongly stimulated the flux of [1,2-^13^C]-glucose into the m + 1 isotopologues of all four ribonucleotides (Fig. 3*C*) indicating that the m+1-ribose-5P was channeled into nucleotide biosynthesis in response to EGF. Furthermore, when MCF-7 cells were labeled with [U-^13^C]-glutamine, EGF stimulated the incorporation of label into the pyrimidine backbones of both UTP and CTP indicating that the cells were actively synthesizing nucleotides *de novo*.

**Figure 4:**
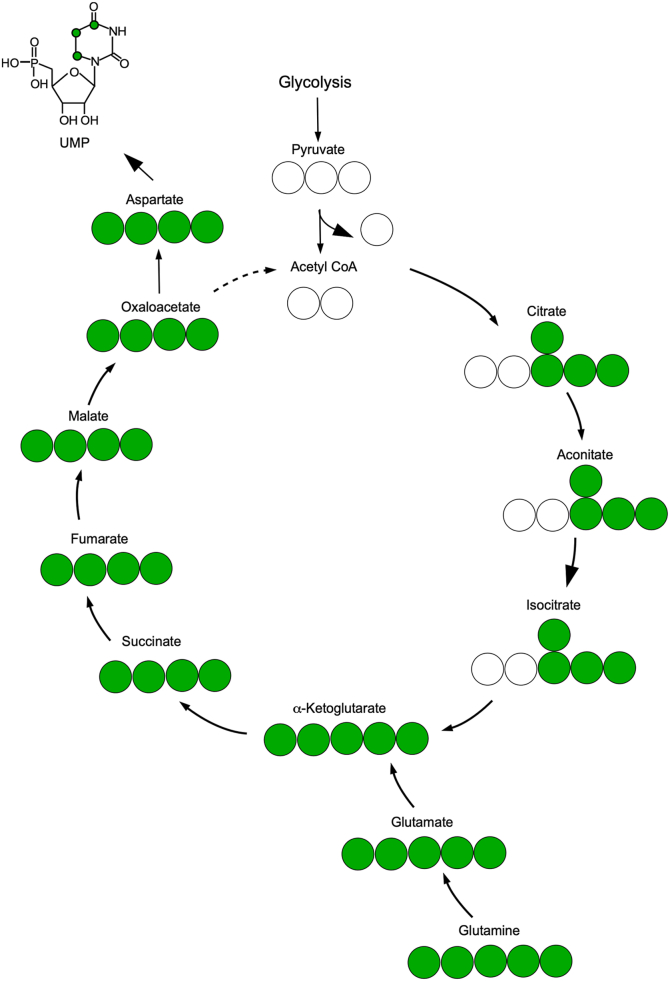
**Flow of ^13^C carbons from [U-^13^C]-glutamine through the TCA cycle and into pyrimidine nucleotide biosynthesis.**^13^C carbons are shown as *green circles*. ^12^C carbons are shown as *open circles*. One ^13^C is lost from m + 5 α-ketoglutarate at the α-ketoglutarate dehydrogenase reaction yielding the m + 4 isotopologs of the subsequent metabolites. One ^13^C carbon is lost from m+4-aspartate in the reactions used for the *de novo* synthesis of pyrimidine nucleotides, yielding the m + 3 isotopologues of UMP and CMP. TCA, tricarboxylic acid.

We examined flux through the TCA cycle with both a [1,2-^13^C]-glucose label and a [U-^13^C]-glutamine label. Figure 4 traces the flow of ^13^C carbons from [U-^13^C]-glutamine through the TCA cycle and into the pyrimidine biosynthetic pathway. As shown in Figure 3*D*, EGF stimulated significant flux of the ^13^C-labeled material from glucose through the TCA cycle as indicated by the increase in enrichment in essentially all of the TCA cycle intermediates after treatment with EGF. Similarly, EGF stimulated the flux of [U-^13^C]-labeled glutamine into almost all of the TCA cycle intermediates (Fig. 3*E*). This suggests that the source of carbons metabolized through the TCA cycle is relatively balanced between glucose-derived and glutamine-derived material.

### The effect of other EGF receptor ligands on metabolism in MCF-7 cells

The EGF receptor has seven different ligands: EGF, TGF*a*, HB-EGF, BTC, AREG, EPG, and EPR. Of these, HB-EGF, BTC, and EPR also bind to ErbB4 whose principal ligands are the neuregulins (NRG) (27). MCF-7 cells express low levels of both the EGF receptor and ErbB4 (Fig. 1) and so would be expected to respond to HB-EGF, BTC, and EPR by activating both the EGF receptor and ErbB4. EGF has been shown to stimulate the proliferation of MCF-7 cells while EPG, EPR, and NRG-2ß stimulate differentiation (22). To determine whether these ligand-specific biological outcomes were associated with differences in the metabolic effects elicited by the ligands, we examined the effect of all seven EGF receptor ligands plus NRG-2ß on metabolism in MCF-7 cells.

**Figure 5:**
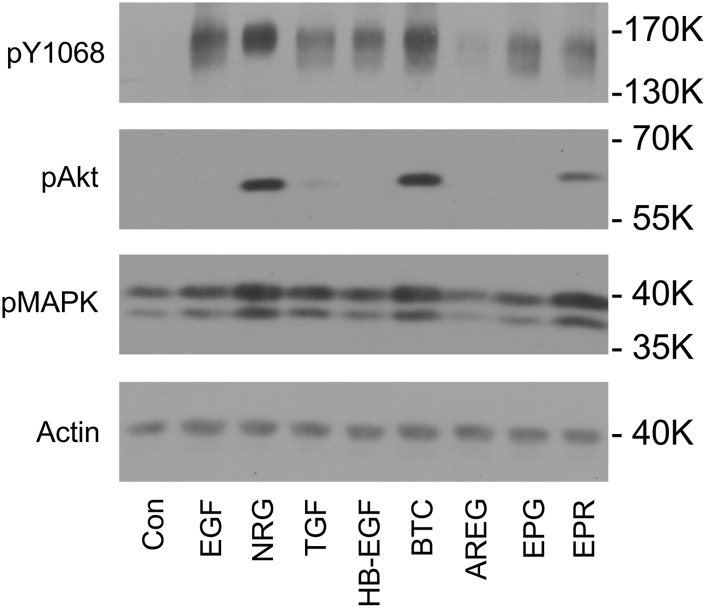
**Agonist-stimulated phosphorylation of the EGF receptor and activation of Akt and MAP kinase in MCF-7 cells.** MCF-7 cells were stimulated for 5 min with the indicated ligands and analyzed by Western blot for stimulation of EGF receptor phosphorylation and activation of Akt or MAP kinase. A total of 60 μg of protein was loaded in each lane. Data are from an experiment that was repeated a total of three times. EGF, epidermal growth factor.

We first tested the ability of the ligands to stimulate the phosphorylation of the EGF receptor and activate Akt and MAP kinase in these cells. As can be seen in Figure 5, NRG-2ß, EGF, and BTC induced the highest level of EGF receptor phosphorylation while TGFα, HB-EGF, EPG, and EPR induced only about half as much. AREG was remarkably weak in stimulating EGF receptor phosphorylation despite being used at its optimal concentration of 300 nM as determined in separate dose response curves. Some of these differences may be due to differential phosphorylation of specific tyrosines in response to a particular ligand.

Despite the fact that seven of the eight ligands tested stimulated strong phosphorylation of the EGF receptor, only NRG-2ß, BTC and EPR stimulated a significant increase in Akt activation. These three ligands also induced the greatest increase in MAP kinase activation. EGF, TGFα, HB-EGF, and EPG also stimulated MAP kinase activation, though to only about a third of the level seen in response to NRG-2ß. AREG was nearly inactive in these assays. Thus, three of the four ligands that bind to ErbB4 induced the strongest effects on Akt and MAP kinase activity in this system. The fourth ligand, HB-EGF, was no better at inducing MAP kinase activation than ligands such as EGF and TGFα that only bind to the EGF receptor. This suggests that the effect of HB-EGF on ErbB4 activation may be qualitatively different from that of NRG-2ß, BTC or EPR.

**Figure 6:**
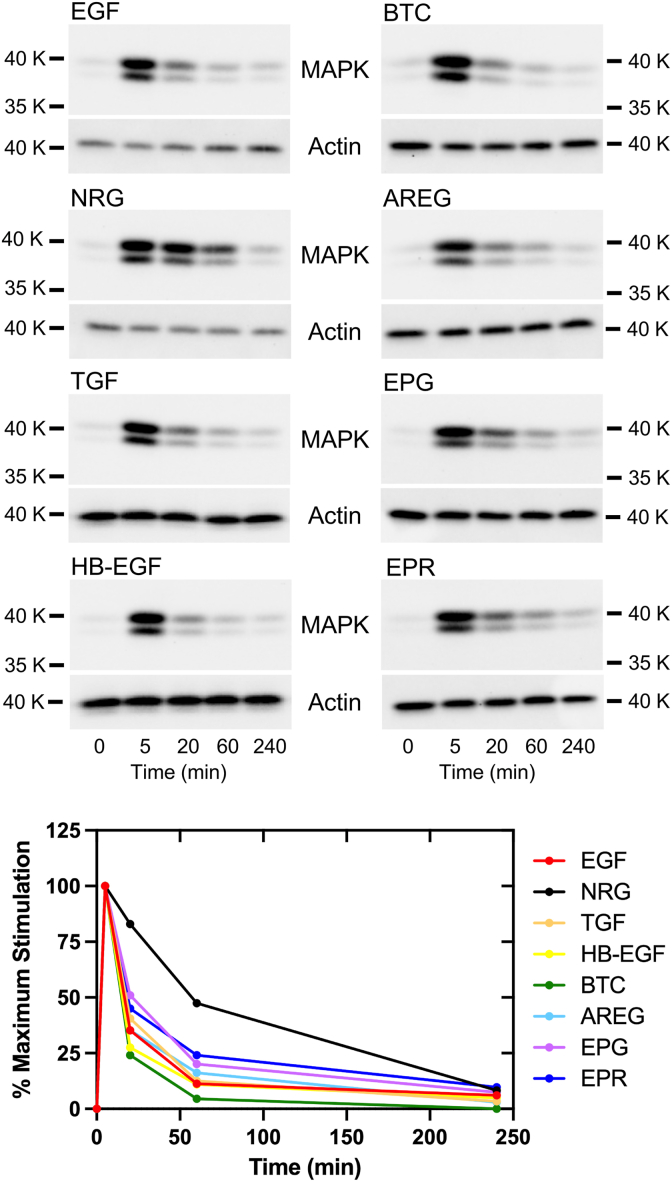
**Time course of MAP kinase activation by multiple agonists in MCF-7 cells.** MCF-7 cells were stimulated with the indicated ligands for times ranging from 0 to 4 h and analyzed by Western blot for stimulation of MAP kinase. The graph shows the results of Image J quantitation of the Western blots. Results for each ligand were normalized to the 5 min time point for that ligand which gave the maximum response for every ligand.

Because EGF receptor ligands have been reported to exhibit differences in the time course of their activation of MAP kinase (22), we assessed this parameter at several time points over a 4-h time course. As shown in Figure 6, activation of MAP kinase peaked at 5 min for all ligands. EGF, TGFα, HB-EGF, and BTC each showed a rapid decline in activation such that by 1 h, MAP kinase stimulation was reduced to ∼10% of the peak level. By contrast, NRG-2ß clearly exhibited a prolonged activation of MAP kinase, retaining 50% of its maximal effect on MAP kinase at 1 h. Consistent with what has been observed previously (22), EPG and EPR retained higher levels of MAP kinase activation at the 20 min and 1 h time points than the high affinity EGF receptor ligands but maintained significantly less MAP kinase activation than NRG-2ß. By 4 h, MAP kinase activation was minimal for all ligands.

**Figure 7:**
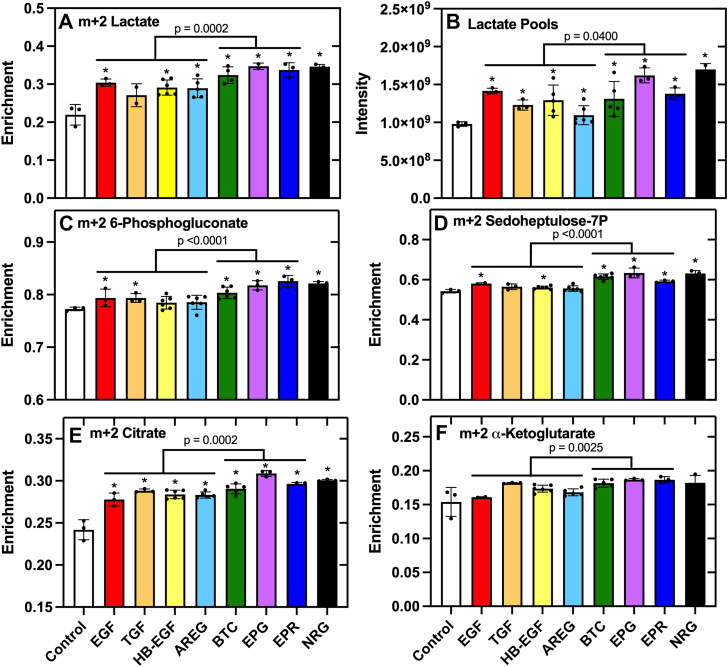
**Effect of multiple agonists on [1,2-^13^C] glucose flux in MCF-7 cells.** MCF-7 cells were labeled with [1,2-^13^C] glucose, stimulated with agonist, and processed for analysis as described in Experimental procedures. Data points represent triplicate or sextuplicate technical replicates from a single biological experiment that was repeated 3 times. Data were tested for statistical significance using unpaired, 2-tailed t-tests. ∗ indicates that the response was significantly different from control at the *p* < 0.05 level. Bars over groups of ligands report the results of the ANOVA analysis.

We next compared the effect of optimal doses of these eight ligands on the flux of [1,2-^13^C]-glucose through the pathways of intermediary metabolism. Figure 7 shows the results for a subset of the main isotopologs of intermediates in glycolysis, the PPP, and the TCA cycle. The ∗ in the figures indicates that the response to that ligand was significantly different from control. In essentially all cases, the EGF receptor ligands modestly increased flux through these major metabolic pathways while NRG-2ß consistently stimulated a much stronger response than EGF. Surprisingly, EPG which only binds to the EGF receptor, generally provoked a response that was equivalent to that of NRG-2ß. The response to EPR, was often significantly higher than the response to EGF though not as high as that for NRG-2ß or EPG. And about half of the time, BTC elicited a response that was significantly greater than that induced by EGF. The responses to EGF, TGFα, HB-EGF, and AREG were largely the same (based on unpaired t-tests). As observed previously with EGF, none of the ligands stimulated a significant increase of flux into ribose-5P and there was no difference among the ligands in the ratio of enrichment in the m + 1, m + 2, and m + 3 isotopologues of ribose-5P (Fig. S2). Thus, the different ligands did not alter the limited use of the nonoxidative pathway of the PPP which allowed the ribose-5P to be used for nucleotide biosynthesis.

These results suggested that there are two kinds of responses to the seven EGF receptor ligands. One response is a moderate stimulation of flux, similar to what is seen with EGF. TGFα, HB-EGF, and AREG also elicit this level of response. The second type of response is a more robust stimulation similar to that provoked by NRG-2ß. EPG, EPR, and BTC fall into this category. To determine whether the difference between the EGF-like and NRG-2ß-like responses was significant, the data for each metabolite were separated into three categories: control, EGF-like (EGF, TGFα, HB-EGF, and AREG) and NRG2ß-like (EPG,EPR, and BTC). The groups were then analyzed using one-way ANOVA followed by a *post hoc* analysis using Tukey’s multiple comparison test. As shown in the panels in Figure 7, the Tukey’s multiple comparison test demonstrated that the response of the NRG-2ß-like group was consistently significantly different from the response to the EGF-like group. The NRG-2ß-like pool, but not the EGF-like pool, was also significantly different from control group.

**Figure 8:**
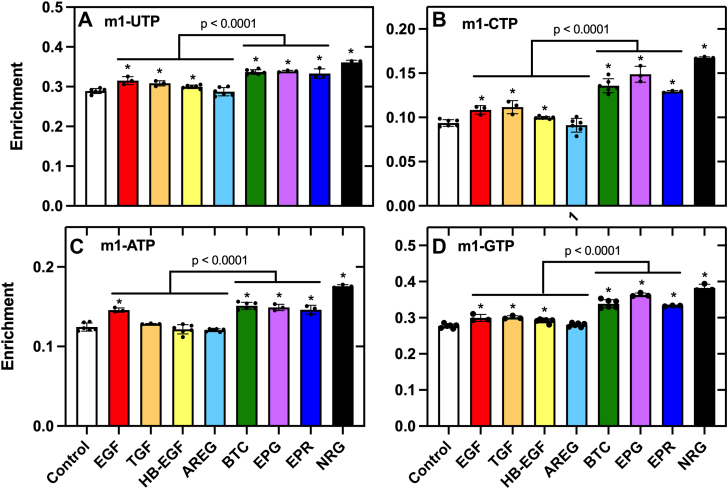
**Effect of multiple agonists on [1,2-^13^C] glucose flux into ribonucleotide triphosphates in MCF-7 cells.** MCF-7 cells were labeled with [1,2-^13^C] glucose, stimulated with agonist, and processed for analysis as described in Experimental procedures. Data points represent triplicate or sextuplicate technical replicates from a single biological experiment that was repeated 3 times. Data were tested for statistical significance using unpaired, 2-tailed t-tests. ∗ indicates that the response was significantly different from control at the *p* < 0.05 level. Bars over groups of ligands report the results of the ANOVA analysis.

Figure 8 shows the effect of these ligands on the flux of [1,2-^13^C]-glucose into ribonucleotides. The m + 1 isotopologues of the NTPs are the result of m + 1 ribose-5P being incorporated into newly synthesized nucleotides. As was true for ligand-stimulated flux through intermediary metabolic pathways, for all four ribonucleotides, NRG-2ß stimulated the highest flux into these isotopologs of any ligand tested. EPR, EPG, and BTC also stimulated significantly more flux into UTP, CTP and GTP than did EGF, TGFα, HB-EGF, and AREG which were generally similar in their effects. Statistical analysis of the data from the EGF-like and NRG-2ß-like groups using ANOVA followed by Tukey’s multiple comparison test again showed that the response to the NRG-2ß-like ligands was consistently significantly different than the response to the EGF-like ligands.

### Comparison with MDA-MB-468 cells

Our observations in MCF-7 cells suggested that the EGF receptor ligands exhibit bias in their ability to stimulate flux through major metabolic pathways. Specifically, BTC, EPG, and EPR were stronger inducers of flux than were EGF, TGF-α, HB-EGF, and AREG. If ligand bias is an intrinsic property of the EGF receptor, then the ligands should exhibit the same bias in all cell types. By contrast, if cell-specific features contribute to differences in signaling, then the same ligands could signal differently in different cells. To address this question, we repeated our experiments in MDA-MB-468 cells, another widely-used breast cancer cell line.

MDA-MB-468 cells differ from MCF-7 cells in their level of expression of ErbB receptors (Fig. 1). Specifically, they overexpress the EGF receptor but lack expression of ErbB4. They also have lower expression of ErbB2 and ErbB3 than do MCF-7 cells. Despite, or perhaps as a result of, the overexpression of the EGF receptor, MDA-MB-468 cells are growth inhibited by EGF (28, 29).

To compare metabolism in MDA-MB-468 cells with that in MCF-7 cells, we again performed metabolic flux experiments in cells labeled with 5 mM [1,2-^13^C]-glucose. As was true in MCF-7 cells, flux into glycolysis and the PPP reached steady state within 20 min (Fig. S3). Flux into the TCA cycle was more rapid than in MCF-7 cells with the reactions of the upper TCA cycle reaching steady state within 20 min and the lower TCA cycle within 2 h. Flux into nucleotides showed a similar time course to that seen in MCF-7 cells but the absolute level of enrichment was significantly lower.

**Figure 9:**
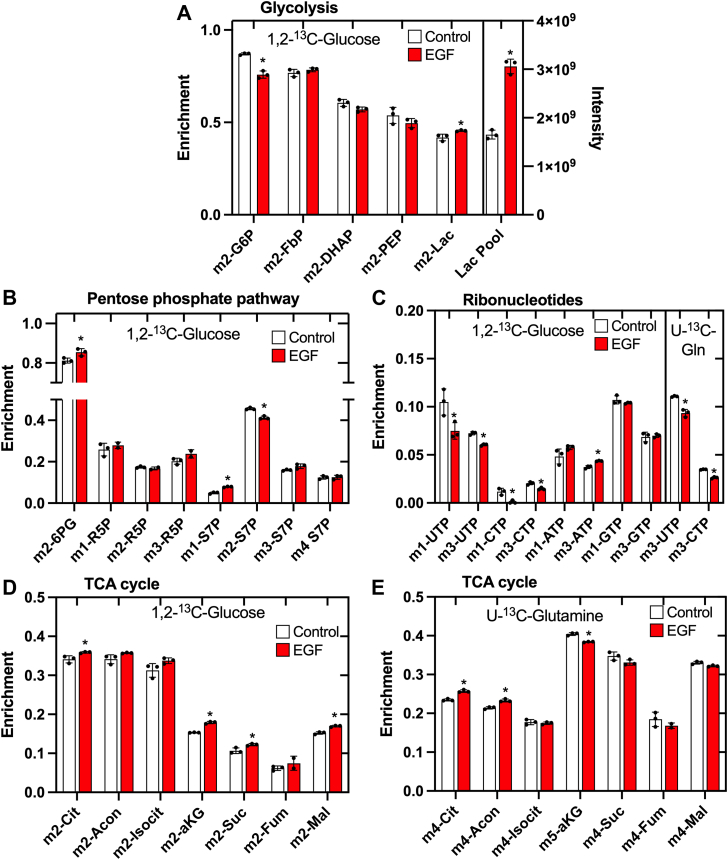
**EGF-stimulated flux of ^13^C-labeled glucose or glutamine through glycolysis, the pentose phosphate pathway, the TCA cycle, and ribonucleotides in MDA-MB-468 cells.***MDA-MB-468* cells were labeled in triplicate with [1,2-^13^C] glucose (panels *A* through *D*) or [U-^13^C] glutamine (panel *E*), stimulated with 30 nM EGF, and extracted for metabolomics analysis as outlined in Experimental procedures. *A*, glycolytic intermediates; *B*, pentose phosphate pathway intermediates; *C*, ribonucleotide triphosphates; *D*, TCA cycle intermediates in cells labeled with [1,2-^13^C] glucose; *E*, TCA cycle intermediates in cells labeled with [U-^13^C] glutamine. Data points represent triplicate technical replicates from a single biological experiment that was repeated twice. ∗ indicates that the difference between control and the EGF-treated sample was significant at the *p* < 0.05 level using unpaired, 2-tailed t-tests. EGF, epidermal growth factor; TCA, tricarboxylic acid.

Figure 9*A* shows the effect of EGF on the flux of 5 mM [1,2-^13^C]-glucose into the major isotopologs of glycolytic intermediates. Surprisingly, EGF inhibited flux into m + 2 glucose-6P, possibly because of an enhanced rate of utilization of the pool of newly generated glucose-6P, as suggested by the stimulation of the flux of label into m + 2 lactate and the significantly increased enrichment of label in the lactate pool. Thus, EGF positively regulates flux through glycolysis in MDA-MB-468 cells.

EGF also regulated flux through the PPP (Fig. 9*B*) by enhancing the entry of glucose into this pathway, as indicated by a significant EGF-stimulated enrichment of label in m + 2 6-phosphogluconate. There was also a significant EGF-induced increase in the m + 1 isotopologue of sedoheptulose-7P but a significant decrease in enrichment in the m + 2 isotopologue of sedoheptulose-7P. Interestingly, the nearly equivalent level of enrichment of label in the m + 1, m + 2, and m + 3 isotopologs of ribose-5P indicates that MDA-MB-468 cells extensively utilize the nonoxidative phase of the PPP to recycle the ribose-5P generated in the oxidative phase of the PPP back into glycolytic intermediates. This recycling of ribose-5P into glycolysis followed by its flow through the glycolytic pathway may account for the decrease in m + 2 sedoheptulose-7P seen in the presence of EGF.

The conclusion that ribose-5P is recycled is supported by the data in Figure 9*C* which show the flux of the ^13^C-label into ribonucleotides. EGF significantly inhibited the enrichment of ^13^C into the m + 1 and m + 3 isotopologs of UTP and GTP. EGF also inhibited the flux of label from U-[^13^C]-glutamine into these pyrimidine nucleotides. Enrichment of the label from [1,2-^13^C]-glucose into purine nucleotides was unaffected by EGF with the exception of the m + 3 isotopologue of ATP which showed a slight enhancement in the presence of EGF. The fact that EGF inhibits the flux of label into nucleotides in MDA-MB-468 cells is consistent with the observation that these cells are growth-inhibited by EGF (28, 29).

In contrast to the lack of EGF stimulation of flux into nucleotides in these cells, the growth factor did stimulate the flux of glucose through the TCA cycle. EGF promoted significant increases in the enrichment of ^13^C from glucose into the m + 2 isotopologues of citrate, alpha-ketoglutarate, succinate, and malate (Fig. 9*D*). The growth factor also stimulated the enrichment of ^13^C from [U-^13^C]-glutamine into m + 4 citrate and m + 4 aconitate (Fig. 9*E*). However, EGF had no effect or actually inhibited the flux of label from [U-^13^C]-glutamine into m + 5 α-ketoglutarate and other lower TCA cycle intermediates. This is consistent with the interpretation that in these cells, EGF preferentially promotes the utilization of glucose as a source of carbons for the TCA cycle. This would result in the dilution of the contribution of carbons from [U-^13^C]-glutamine which enters at a lower point in the TCA cycle.

### The effect of other EGFR ligands on metabolism in MDA-MB-468 cells

We next compared the effect of optimal concentrations of the seven EGF receptor ligands plus NRG-2ß on EGF receptor phosphorylation and activation of Akt and MAP kinase in MDA-MB-468 cells. MDA-MB-468 cells express very high levels of EGF receptors and no detectable ErbB4. Thus, even the EGF receptor ligands that can bind to ErbB4 (HB-EGF, BTC, and EPR) cannot activate that receptor, so the signaling response is dominated by the EGF receptor. This differs from the situation in MCF-7 cells where both the EGF receptor and ErbB4 were expressed and could be activated by the ligands with overlapping binding specificity.

**Figure 10:**
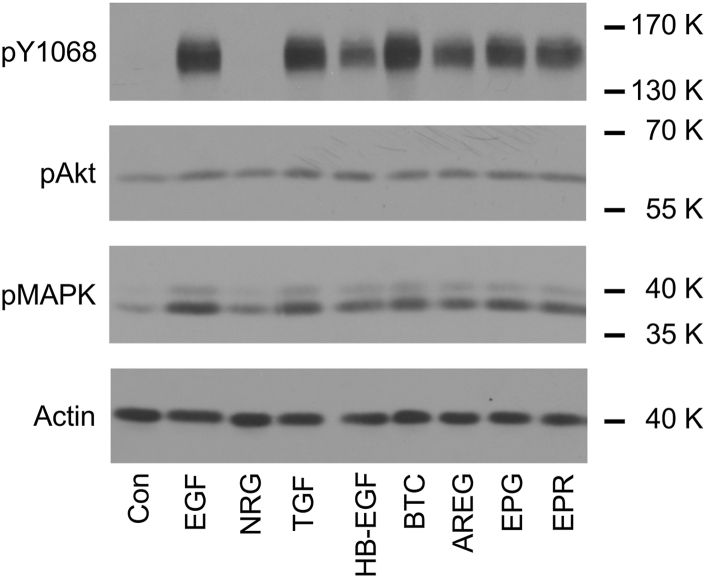
**Agonist-stimulated phosphorylation of the EGF receptor and activation of Akt and MAP kinase in MDA-MB-468 cells.***MDA-MB-468* cells were stimulated for 5 min with the indicated ligands and analyzed by Western blot for phosphorylation of the EGF receptor and for activation of Akt or MAP kinase. A total of 60 μg of protein was loaded in each lane. Data are from an experiment that was repeated twice. EGF, epidermal growth factor.

As shown in Figure 10, in MDA-MB-468 cells, all of the EGF receptor-binding ligands induced robust phosphorylation of the EGF receptor while NRG-2ß did not. All EGF receptor-binding ligands also stimulated the activation of MAP kinase with EGF, and TGFα showing slightly greater activation than the other ligands. A similar pattern is seen for the activation of Akt, though basal activity was significant, and the absolute level of stimulated activity was quite low, making accurate quantitation difficult. NRG-2ß induced activation of both Akt and MAP kinase, but it was clearly the weakest agonist among the eight tested. These results contrast with those seen in MCF-7 cells in which NRG, BTC, and EPR showed significantly greater efficacy for stimulating MAP kinase and Akt than did the five ligands that bind only to the EGF receptor. In addition, AREG was quite weak in MCF-7 cells but showed good efficacy in both the Akt and MAP kinase assays in MDA-MB-468 cells. Thus, the pattern of ligand efficacy was different in the two cell lines.

**Figure 11:**
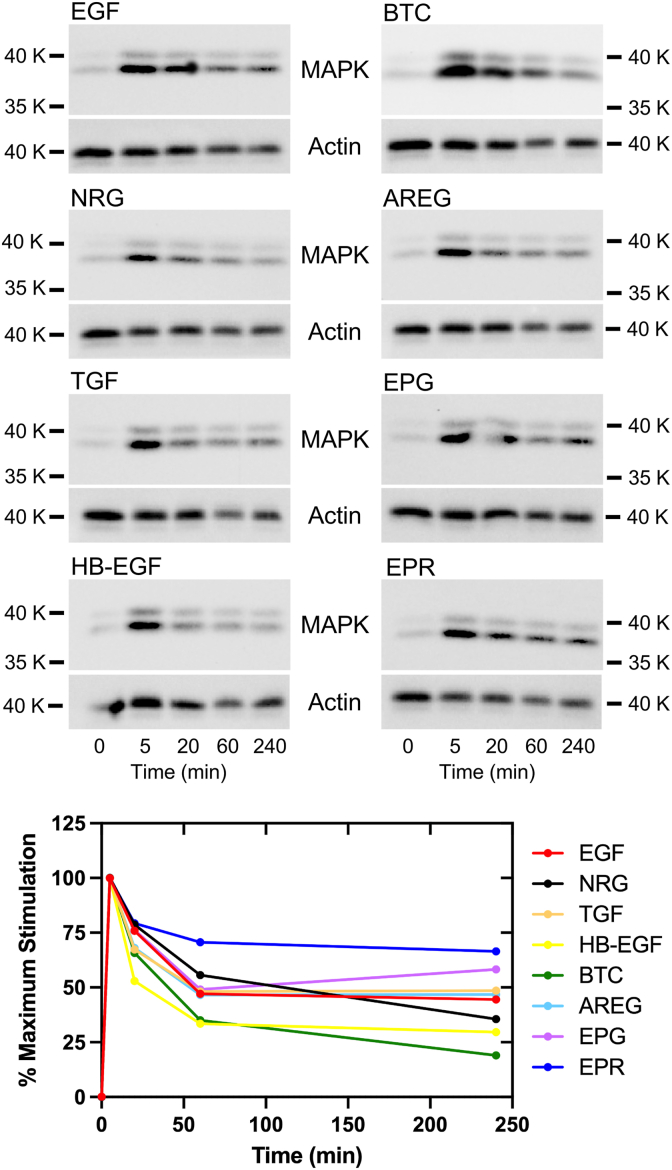
**Time course of MAP kinase activation by multiple agonists in MDA-MB-468 cells.** MDA-MB-468 cells were stimulated with the indicated ligands for times ranging from 0 to 4 h and analyzed by Western blot for stimulation of MAP kinase. The graph shows the results of Image J quantitation of the Western blots. Results for each ligand were normalized to the 5 min time point for that ligand which gave the maximum response for every ligand.

In experiments examining the time course of activation of MAP kinase, all of the ligands elicited a more sustained response in MDA-MB-468 cells than was observed in MCF-7 cells (Fig. 11). But there was more variation among the different ligands. The average stimulation of all eight ligands at 1 h was ∼50% of the peak response, but EPR retained 70% of its peak response while HB-EGF and BTC retained only ∼35% of their peak response at 1 h. The significantly more prolonged response to agonist ligands in these cells as compared to MCF-7 cells could reflect differences in the level of expression of phosphotyrosine phosphatases and their regulation by EGF receptor ligands.

**Figure 12:**
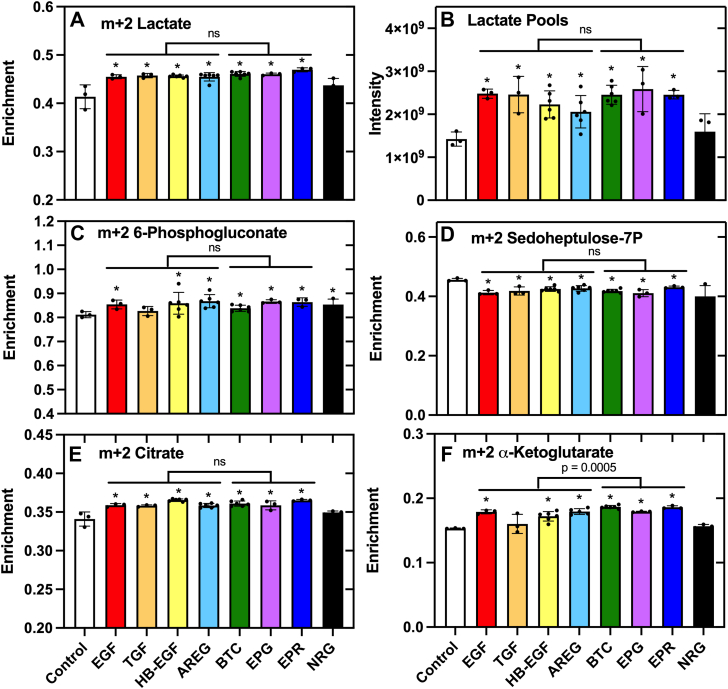
**Effect of multiple agonists on [1,2-^13^C] glucose flux in MDA-MB-468 cells.***MDA-MB-468* cells were labeled with [1,2-^13^C] glucose, stimulated with agonist, and processed for analysis as described in Experimental procedures. Data points represent triplicate or sextuplicate technical replicates from a single biological experiment that was repeated 3 times. Data were tested for statistical significance using unpaired, 2-tailed t-tests. ∗ indicates that the response was significantly different from control at the *p* < 0.05 level. Bars over groups of ligands report the results of the ANOVA analysis.

Figure 12 shows the effect of optimal doses of these eight ligands on the flux of [1,2-^13^C]-glucose into a subset of the main isotopologs of intermediates in glycolysis, the PPP, and the TCA cycle. As before, the ∗ indicates that a response is statistically significantly different from the control response. The seven ligands that bound to the EGF receptor all stimulated flux into m + 2 lactate to the same extent and increased the pools of lactate to roughly the same levels. By contrast, NRG-2ß failed to stimulate either of these responses. Similarly, the effects of the seven EGF receptor-binding ligands on flux into the intermediates of the PPP (m + 2 6-phosphogluconate, m + 2 sedoheptulose-7P) and the TCA cycle (m + 2 citrate and m + 2 alpha-ketoglutarate) were largely the same in all cases and NRG-2ß again failed to modulate any of these fluxes. None of the ligands induced a difference in the utilization of the nonoxidative reactions of the PPP as judged by the similarity of flux into the isotopologues of ribose-5P (Fig. S4).

To determine whether there was a difference in the response to the EGF-like and NRG-2ß-like ligands in MDA-MB-468 cells, we subjected these data to the same ANOVA/Tukey’s analysis that was performed on the data from MCF-7 cells. As shown in Figure 12, in MDA-MB-468 cells, with the exception of α-ketogluarate, the responses to the EGF-like and NRG-2ß-like ligands were not significantly different from each other. This contrasts with what was observed in MCF-7 cells in which the response to the NRG-2ß-like ligands was consistently significantly different from the response to the EGF-like ligands. Similarly, the responses to both the EGF-like and NRG-2ß-like ligands were significantly different from the control in MDA-MB-468 cells whereas only the NRG-2ß-like responses were different from control in MCF-7 cells.

**Figure 13:**
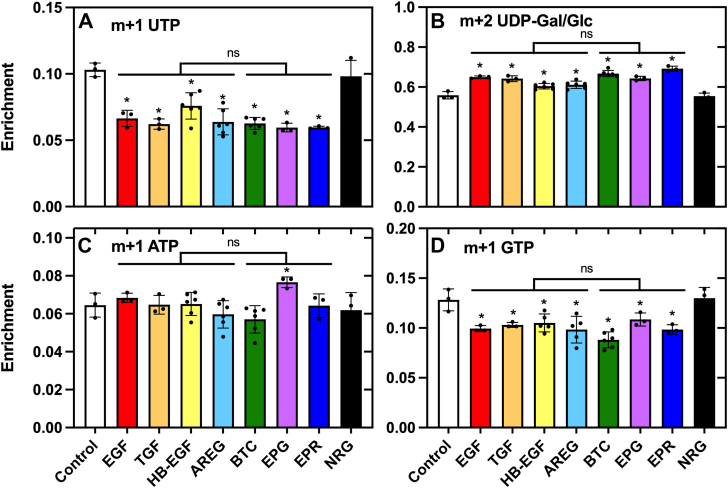
**Effect of multiple agonists on [1,2-^13^C] glucose flux into ribonucleotide triphosphates in MCF-7 cells.***MDA-MB-468* cells were labeled with [1,2-^13^C] glucose, stimulated with agonist, and processed for analysis as described in Experimental procedures. Data points represent triplicate or sextuplicate technical replicates from a single biological experiment that was repeated 3 times. Data were tested for statistical significance using unpaired, 2-tailed t-tests. ∗ indicates that the response was significantly different from control at the *p* < 0.05 level. Bars over groups of ligands report the results of the ANOVA analysis.

A similar pattern was seen for the effects of these ligands on flux of ^13^C into nucleotides (Fig. 13). EGF and the other EGF receptor-binding ligands inhibited flux into m + 1 UTP and m + 1 GTP while the response to NRG-2ß was not different from the control. With the exception of EPG, none of the ligands elicited a significant effect on flux into m + 1 ATP. No flux into m + 1 CTP was detected in these cells. However, the seven EGF receptor ligands did stimulate the flux of label into m + 2 UDP-Glc/Gal suggesting that the input [1,2-^13^C]-glucose may have reacted with the preexisting pool of unlabeled UTP to form this biosynthetic intermediate. This may be relevant to the known ability of these cells to carry out glycogen synthesis (30). Statistical analysis of these data again showed no difference in the response to the EGF-like and NRG-2ß-like responses.

## Discussion

### Differences in metabolism in MCF-7 and MDA-468 cells

EGF stimulates the proliferation of MCF-7 cells (22) but inhibits the growth of MDA-MB-468 cells (28, 29). Nonetheless, both cell lines responded to EGF with an increase in flux through the early pathways of intermediary metabolism. A notable difference between the two cell lines was that MCF-7 cells preferentially channeled ribose-5P into nucleotide biosynthesis while MDA-MB-468 cells utilized the reactions of the nonoxidative phase of the PPP to recycle ribose-5P into glycolytic intermediates. This distinction was apparent even in the absence of growth factor, indicating that the two cell lines differ in how they regulate glycolysis and the PPP under basal conditions. As a result of these differences, EGF stimulated enhanced flux of ^13^C into nucleotides in MCF-7 cells but actually inhibited the flux of ^13^C into nucleotides in MDA-MB-468 cells. Thus, by 4 h poststimulation, the two lines already showed evidence of their divergence into a growth promoting *versus* a growth inhibitory program.

The marked channeling of PPP intermediates back into glycolysis in MDA-MB-468 cells is consistent with our observation that these cells appear to preferentially use carbons sourced from glucose rather than glutamine in the TCA cycle. MCF-7 cells appear to utilize a balance of glucose and glutamine to feed their TCA cycle. These findings are similar to those reported by Ripoll *et al.* (31).

### Differences in ligand bias in MCF-7 and MDA-468 cells

Comparison of the metabolic effects of the seven EGF receptor ligands plus NRG-2ß in MCF-7 cells and MDA-MB-468 cells revealed differences in the cells’ response to the various agonists. In MCF-7 cells, the data suggested the presence of two classes of response to EGF receptor ligands: a more robust, “NRG-like” response provoked by BTC, EPR, and EPG and a more modest “EGF-like” response elicited by EGF, TGFα, HB-EGF, and AREG. By contrast, in MDA-MB-468 cells, the seven EGF receptor ligands all elicited a similar response so there was only an “EGF-like” response. The differences between the responses to the EGF receptor ligands in these two cell lines suggest that there is ligand bias in MCF-7 cells but no ligand bias in MDA-MB-468 cells.

Bias in the EGF receptor system has been linked to differences in the conformation of the dimerized extracellular domain of the EGF receptor, with high affinity ligands, such as EGF (3) and TGFα (2), inducing a symmetric extracellular domain dimer and low affinity ligands inducing an asymmetric dimer (22). EGF has also been shown to induce dimers that are more stable than those formed in response to the binding of the low affinity ligands, EPG and EPR (22). In cryo-EM structures, EGF induced a conformation in which the tips of subdomain IV of the extracellular domain were separated by a greater distance than when TGFα was bound to the receptor (32). Schepartz and colleagues (33, 34) used bipartite tetracysteine display to show that EGF and HB-EGF induced the formation of an antiparallel coiled coil of the intracellular juxtamembrane domain of the EGF receptor while TGFα, AREG, EPR, and EPG induced an alternate helical dimer and BTC induced an intermediate structure. Thus, ligand-induced conformational differences in the extracellular domain are associated with differences in the conformation of the intracellular juxtamembrane domain. These differences lead to alterations in the sites of autophosphorylation in the C-terminal tail (24, 35, 36) and the overall level of ligand-induced receptor tyrosine phosphorylation (32, 37, 38).

All of these ligand-specific differences suggest that ligand bias is an intrinsic property of the EGF receptor. That is, bias is due to ligand-induced changes within the receptor structure that modify the signaling output of the receptor itself. If ligand bias were solely dependent on intrinsic properties of the EGF receptor, then one would expect that the relative efficacy of the seven ligands for eliciting a biological response would be consistent across all systems. Our data demonstrate that this is not the case as all ligands show equal efficacy in MDA-MB-468 cells but different efficacies in MCF-7 cells. This implies that a cell-intrinsic property contributes to the variation in ligand responses.

In this case, the relevant cell-intrinsic feature is likely to be the expression of other ErbB family members. We favor ErbB4 as the main contributor to the observed differences in ligand response between MCF-7 and MDA-MB-468 cells. MDA-MB-468 cells express extremely high levels of EGF receptors with only low levels of ErbB2 and ErbB3 and no ErbB4. Thus, the EGF receptor strongly dominates the ErbB signaling environment. By contrast, MCF-7 cells express low levels of the EGF receptor and ErbB4 and somewhat higher levels of ErbB2 and ErbB3. They therefore have a more diverse ErbB signaling complement. In these cells, ligands such as BTC, HB-EGF, and EPR that bind to both the EGF receptor and ErbB4 could potentially activate pathways not available to ligands that bind only to the EGF receptor.

We speculate that the response to any particular EGF receptor ligand is determined, not simply by the EGF receptor, but by the entire assemblage of ErbB receptors present in the cell. MDA-MB-468 cells vastly overexpress EGF receptors, yet have few other ErbB receptors. Thus, the vast majority of active dimers in the ligand-stimulated state will be EGF receptor homodimers. The output from stimulation by EGF receptor ligands would therefore almost exclusively reflect the signaling properties of the EGF receptor. This would give rise to the “EGF-like” response of all ligands seen in MDA-MB-468 cells. The fact that NRG-2ß, an ErbB3-ErbB4 ligand, did not stimulate changes in metabolic flux is consistent with a minimal contribution of ErbB3 or ErbB4 to signaling in these cells.

In MCF-7 cells that express similar, low levels of EGF receptors and ErbB4, a mixture of EGF receptor homodimers or EGFR/ErbB4 heterodimers would form in response to agonists that bind only to the EGF receptor. Due to the presence in these cells of relatively higher levels of ErbB2 and ErbB3, heterodimers containing ErbB2 or ErbB3 could also form, though EGFR/ErbB3 heterodimers tend to be disfavored (18, 39). For agonists such as BTC and EPR that bind to both the EGFR and ErbB4, homodimers of the EGF receptor or ErbB4, as well as heterodimers of EGFR/ErbB2, EGFR/ErbB4, ErbB2/ErbB4, or Erb3/ErbB4 could form, generating a signal that reflects the output from a mixture of the different dimers. It is possible that a ligand that is capable of inducing the formation of dimers containing ErbB4 gives rise to the “NRG-like” response seen for some ligands in MCF-7 cells, whereas a ligand that mainly induces dimers that contain the EGF receptor might give rise to the “EGF-like” response seen for the other ligands in these cells.

Of the EGF receptor-binding ligands, BTC, EPR, and EPG generated a “NRG-like” response in MCF-7 cells. BTC and EPR bind to both the EGF receptor and ErbB4. As a result, they would be able to induce the formation of homo- or hetero-dimers containing the EGF receptor or ErbB4. However, EPG binds only to the EGF receptor (40). Thus, its capacity to induce a “NRG-like” response cannot be due to a direct interaction with ErbB4. In solution, EPG stabilizes a conformation of the extracellular domain of the EGF receptor [20] that is similar to that of constitutively-open ErbB2 (41). ErbB2 does not homodimerize due to a failure to form an appropriate dimerization arm binding pocket (42). Hence, it is an obligate heterodimerization partner. The EPG-bound EGF receptor may be functionally similar to ErbB2, disfavoring EGF receptor homodimerization if a heterodimerization partner is present. Thus, EPG could drive the EGF receptor toward heterodimerization with ErbB3 or ErbB4 in MCF-7 cells to yield the “NRG-like” response.

Although HB-EGF binds to both the EGF receptor and ErbB4, it elicited an “EGF-like” response in MCF-7 cells. It is possible that HB-EGF preferentially induces EGF receptor homodimers and fails to induce ErbB4 homodimers or ErbB3/ErbB4 heterodimers, despite being able to bind to ErbB4. The predominance of EGF receptor homodimers would yield the “EGF-like” response. This possibility is consistent with the observations of Beerli *et al.* (43) who reported that while NRG and BTC strongly stimulated phosphorylation of ErbB4, HB-EGF induced only a weak response.

EPG and EPR, but not EGF, stimulate the differentiation of MCF-7 cells (22, 44). The ability of these EGF receptor-binding ligands to stimulate differentiation has been linked to the fact that they exhibit a low affinity for the EGF receptor, form asymmetric, less stable, extracellular domain dimers and generate a more persistent activation of MAP kinase than does EGF (22). The observation that EPG and EPR induce a “NRG-like” metabolic response, is consistent with the fact that NRG also stimulates differentiation in MCF-7 cells. However, BTC, a high affinity ligand that presumably forms stable dimers, and induces only transient activation of MAP kinase in MCF-7 cells (Fig. 6 and (22)), also induced a “NRG-like” metabolic effect. Thus, the distinction between stable *versus* unstable dimers and transient *versus* persistent activation of MAP kinase may not be central to the ability of a ligand to induce differentiation of MCF-7 cells. The differences may relate more to their ability to drive the choice of dimerization partner.

The notion that there is differential dimerization among the ErbB family members is not new (17, 18, 45). However, the role of ligands in the selection of dimerization partners has only recently begun to come into focus. EGF only weakly stimulates EGFR/ErbB4 heterodimerization while NRG1 strongly promotes such heterodimerization (46). Both NRG1 and BTC have been found to preferentially induce formation of ErbB4 homodimers over ErbB2/ErbB4 heterodimers (19). Using luciferase fragment complementation, we have shown that AREG showed lower efficacy for inducing EGF receptor homodimers or EGFR/ErbB2 heterodimers than did EGF, TGFα, or BTC (38). And BTC, but not EGF, induced the formation of EGFR/ErbB3 heterodimers and ErbB3 phosphorylation in hTCEpi cells (37). Thus, accumulating evidence suggests that different ligands promote differential dimerization of ErbB receptors. Signaling in the ErbB system may therefore be the reflection of a complex equilibrium, the position of which is determined by the absolute level of expression of all ErbB receptors, the affinity of one ErbB receptor for another ErbB receptor, the affinity of a given ligand for each receptor, and the ability of a ligand to preferentially promote dimerization of selected ErbB receptors. Our findings here highlight the need to consider the relative level of expression of all ErbB receptors in cells when interpreting data related to ligand bias. If ligand bias involves the preferential induction of certain dimers, it may only be apparent in a system if the relevant ErbB receptors are present.

## Experimental procedures

### Materials

MCF-7 and MDA-MB-468 cells were obtained from the American Type Culture Collection. EGF and AREG were from Gold Biotechnology. EPG, EPR, and BTC were from Prospec. HB-EGF was from Sigma-Aldrich. TGFα was from Abcam. NRG-2ß was from R&D Systems. The EGF receptor antibody (cat. #2232L, lot 15), ErbB4 antibody (cat. #4795s, lot 7), and the pY1068 EGF receptor antibody (cat. #2234S, lot 13) were from Cell Signaling Technology. The C-terminal ErbB2 (cat. #06-562, lot 2477830) and ErbB3 (cat. #05-390, lot 2000800) antibodies were from Millipore. The phospho-Akt (cat. #4060S, lot 27) and phospho-MAPK (cat. #9101S, lot 32) antibodies were also from Cell Signaling Technology. The actin antibody (cat. # 1501, lot 81988) was from Chemicon International. Peroxidase-conjugated sheep anti-mouse IgG was from Rockland (cat. #610-603-002). Peroxidase-conjugated donkey anti-rabbit IgG was from Thermo Fisher Scientific (cat. #31458). ECL Western blotting substrate was from Pierce (cat. # 32106) or Immobilon Western chemiluminescent HRP substrate was from Millipore (cat. # WBKLS). [1,2-^13^C]-glucose and U-^13^C-glutamine were obtained from Cambridge Isotope Laboratories, Inc. Zymo DNA/RNA Shield was from Plasmidsaurus.

### Cells and tissue culture

MCF-7 cells were maintained in Dulbecco’s modified Eagle medium containing 10% fetal bovine serum and 10,000 U/ml penicillin/streptomycin. MDA-MB-468 cells were maintained in high glucose Dulbecco’s modified Eagle medium containing 10% fetal bovine serum and 10,000 U/ml penicillin/streptomycin.

For metabolomics experiments, cells were plated in triplicate or sextuplicate onto 6-well dishes 24 h prior to use. Approximately 16 h before use, cells were changed into fresh media, containing 5 mM glucose with 2 mM glutamine. At t = 0, cells were washed three times with warm phosphate-buffered saline and Dulbecco’s modified Eagle medium containing 5 mM [1,2-^13^C]-glucose or 2 mM [U-^13^C]-glutamine was added in the absence or presence of the indicated ligand. Ligand concentrations were: EGF, 30 nM; TGFα, 100 nM; BTC, 100 nM; HB-EGF, 30 nM; AREG, 300 nM; EPG, 5 μM, EPR, 5 μM, and NRG-2ß, 10 nM. After incubation for 4 h at 37 °C, monolayers were processed for metabolomic analysis as described previously (47).

For receptor phosphorylation, Akt and MAPK activation, cells were plated into 6-well dishes 48 h before use. Cells were switched to Dulbecco’s modified Eagle medium containing 5 mM glucose 16 h prior to use. Cells were stimulated with the concentration of growth factor noted above for 5 min at 37 °C, after which monolayers were washed with ice-cold phosphate-buffered saline and RIPA lysates were prepared. Equal amounts of total protein were analyzed by SDS polyacrylamide gel electrophoresis and subsequently blotted for phosphotyrosine, phospho-Akt, phospho-MAP kinase, or actin. Horseradish peroxidase-conjugated secondary antibodies were used to detect the primary antibodies. Bands were visualized using MidSci Classic Blue X-ray film developed in a Konica Minolta SRX-101A film processor. For direct imaging, a Bio-Rad ChemiDoc MP Imaging System was used.

For the time course of MAP kinase activation, cells were plated into 6-well dishes 48 h before use and switched into Dulbecco’s modified Eagle medium containing 0.1% fetal calf serum prior to use. Cells were stimulated with 30 nM EGF for times ranging from 0 to 4 h and processed as above. Bands on Western blots were quantitated using Image J software for densitometry. After quantitation of bands, the density associated with the control lane was subtracted from the density in the hormone-stimulated lanes. This yielded the ligand-stimulated response for each hormone. The highest stimulated response, which occurred at 5 min, was set to 100 and the responses at other time points for that hormone were normalized to that response.

### LC-MS analysis

Ultra-high-performance liquid chromatography–mass spectrometry (UHPLC–MS) analyses were performed using a Thermo Scientific Vanquish Flex UHPLC system coupled to a Thermo Scientific Orbitrap ID-X mass spectrometer. Polar metabolites were separated on a HILICON iHILIC-(P) Classic HILIC column (100 × 2.1 mm, 5 μm) with a guard column (20 × 2.1 mm, 5 μm). The mobile phases consisted of solvent A (20 mM ammonium bicarbonate, 2.5 μM medronic acid, and 0.1% ammonium hydroxide in 95:5 water:acetonitrile) and solvent B (95:5 acetonitrile:water). The column temperature was maintained at 45 °C, and separations were performed at a flow rate of 250 μl/min using the following linear gradient: 90% B from 0 to 1 min; decreased to 35% B at 12 min; further decreased to 25% B from 12.5 to 14.5 min; and returned to 90% B at 15 min for column reequilibration. Data were acquired in both positive and negative electrospray ionization modes. Raw LC–MS data were processed and analyzed using X13CMS, Compound Discoverer, Skyline, and AccuCor software packages (48). Data were analyzed statistically *via* unpaired, two-tailed student t-tests in Excel or one-way ANOVA followed by Tukey’s multiple comparisons test in GraphPad Prism 10. Data were plotted using GraphPad Prism 10.

### RNA-seq

MCF-7 or MDA-MB-468 cells were plated and grown as indicated above. Monolayers were washed with PBS, trypsinized, and cells counted. Approximately 2 × 10^5^ cells were pelleted and resuspended in 50 μl Zymo DNA/RNA Shield. Zymo DNA/RNA Shield immediately lyses the cells and stabilizes the RNA for up to 30 days at RT. Samples were submitted for RNA Seq analysis by Plasmidsaurus. Because they use 3′ end counting, there is no transcript length bias and the number of reads is directly related to the number of transcripts. Technical details of the RNA-Seq analysis carried out by Plasmidsaurus may be found on their website (https://plasmidsaurus.com/technical-documentation/rna).

## Data availability

All data supporting the findings of this study are included within the article and its supporting information files. Raw experimental data are available from the corresponding author upon reasonable request.

## Supporting information

This article contains Supporting Information Figs. S1 through S4.

## Conflict of interest

G. J. P. has a collaborative research agreement with Thermo Fisher Scientific, and G. J. P. is a scientific advisory board member for Cambridge Isotope Laboratories and the chief scientific officer of Panome Bio. The other authors declare no conflicts of interest.
