# Supplementary material for: Differential ErbB receptor dimerization modulates the ability of EGF receptor ligands to regulate metabolic flux
Source: J Biol Chem. 2026 Jun 4;302(7):113230. doi: 10.1016/j.jbc.2026.113230 (PMC13330678; doi:10.1016/j.jbc.2026.113230)
Supplement: Supporting Information [file mmc1.docx]

Supporting information for:

Differential ErbB receptor dimerization modulates

the ability of EGF receptor ligands to regulate metabolic flux

Jennifer Macdonald-Obermann^1^, Kevin Cho^2^, Gary J. Patti^2^, Linda J. Pike^1^

Figure S1

Figure S2

Figure S3

Figure S4

**Supporting Information**

**Figure S1**


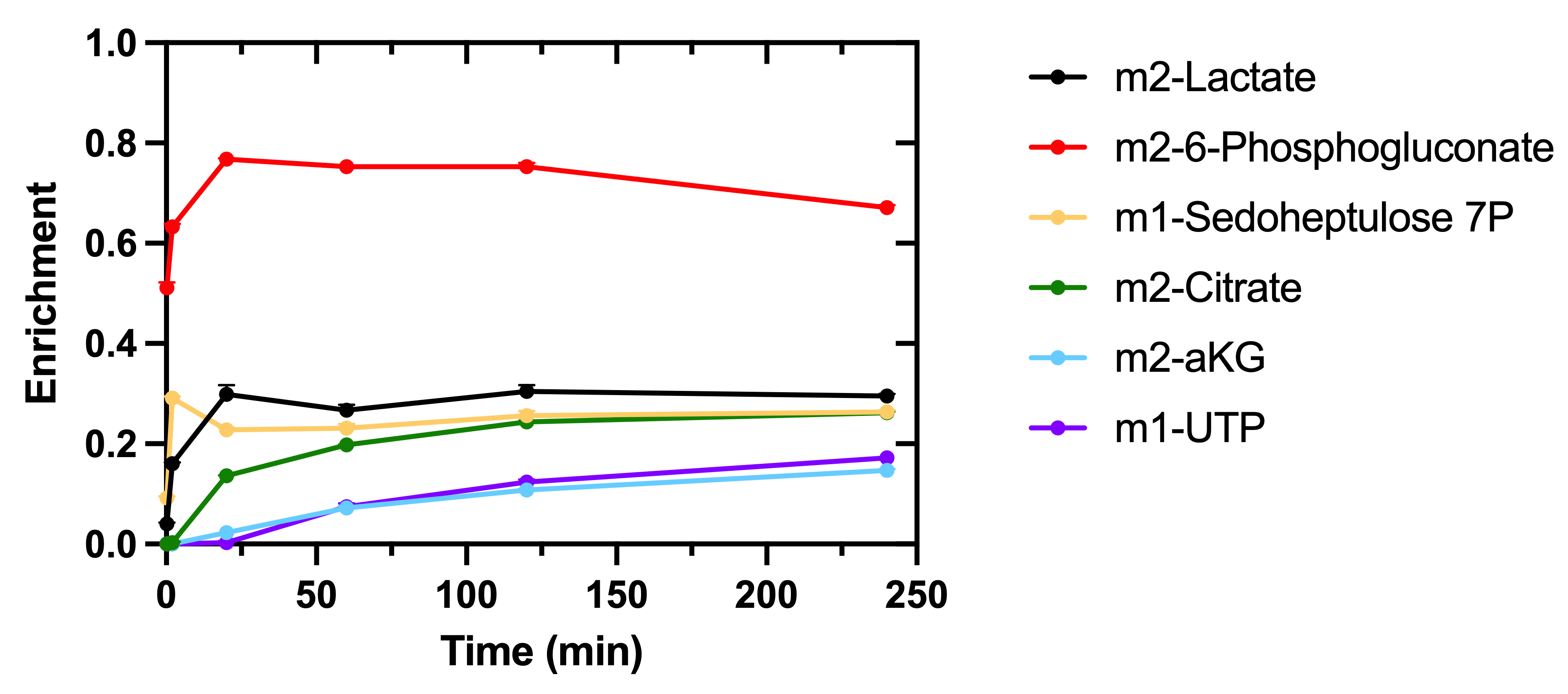


**Figure S1**. *Time course of the flux of [1,2-^13^C]-glucose into representative intermediates of metabolic pathways in MCF-7 cells*. MCF-7 cells were labeled with [1,2-^13^C] glucose for 15 sec, 2 min, 20 min, 60, min, 2 hr, and 4 hr, and extracted for metabolomics analysis as outlined in Experimental Procedures. Points represent the mean +/- standard deviation of triplicate determinations.

**Supporting Information**

**Figure S2**


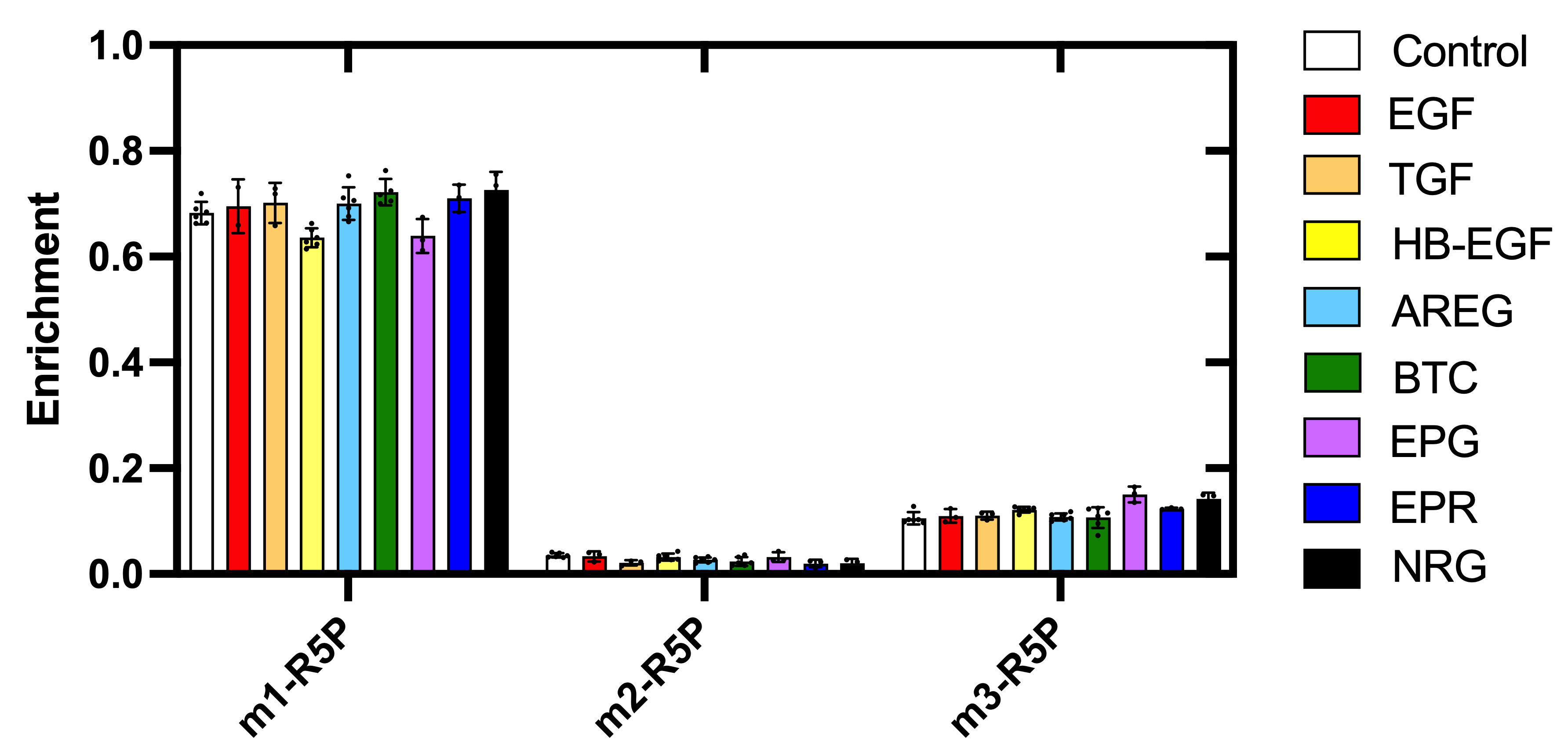


**Figure S2**. *Flux of [1,2-^13^C]-glucose into isotopologues of ribose-5P in MCF-7 cells*. MCF-7 cells were labeled with [1,2-^13^C] glucose, stimulated with ligand and processed for metabolomics analysis as outlined in Experimental Procedures. Bars represent the mean +/- standard deviation of triplicate or sextuplicate determinations.

**Supporting Information**

**Figure S3**


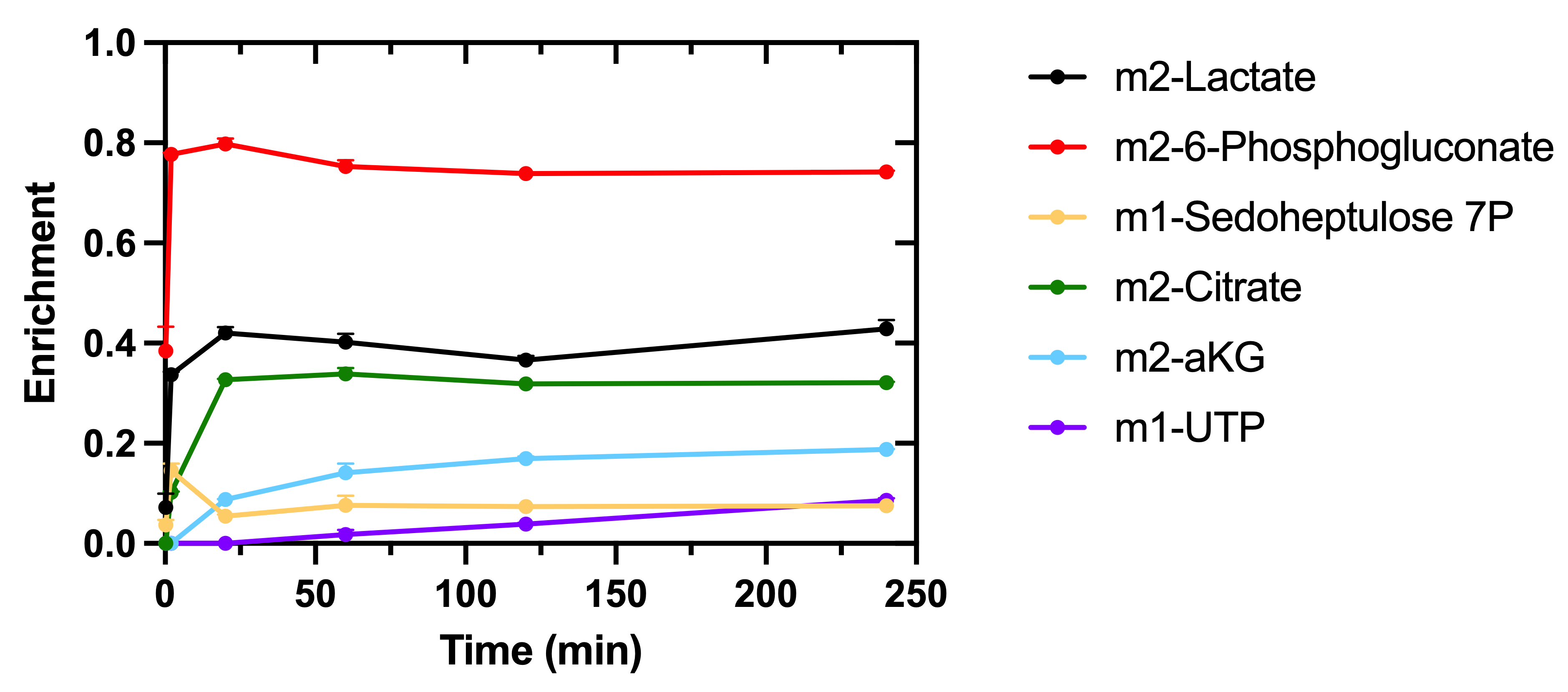


**Figure S3**. *Time course of the flux of [1,2-^13^C]-glucose into representative intermediates of metabolic pathways in MDA-MB-468 cells*. MDA-MB-468 cells were labeled with [1,2-^13^C] glucose for 15 sec, 2 min, 20 min, 60, min, 2 hr, and 4 hr, and extracted for metabolomics analysis as outlined in Experimental Procedures. Points represent the mean +/- standard deviation of triplicate determinations.

**Supporting Information**

**Figure S4**


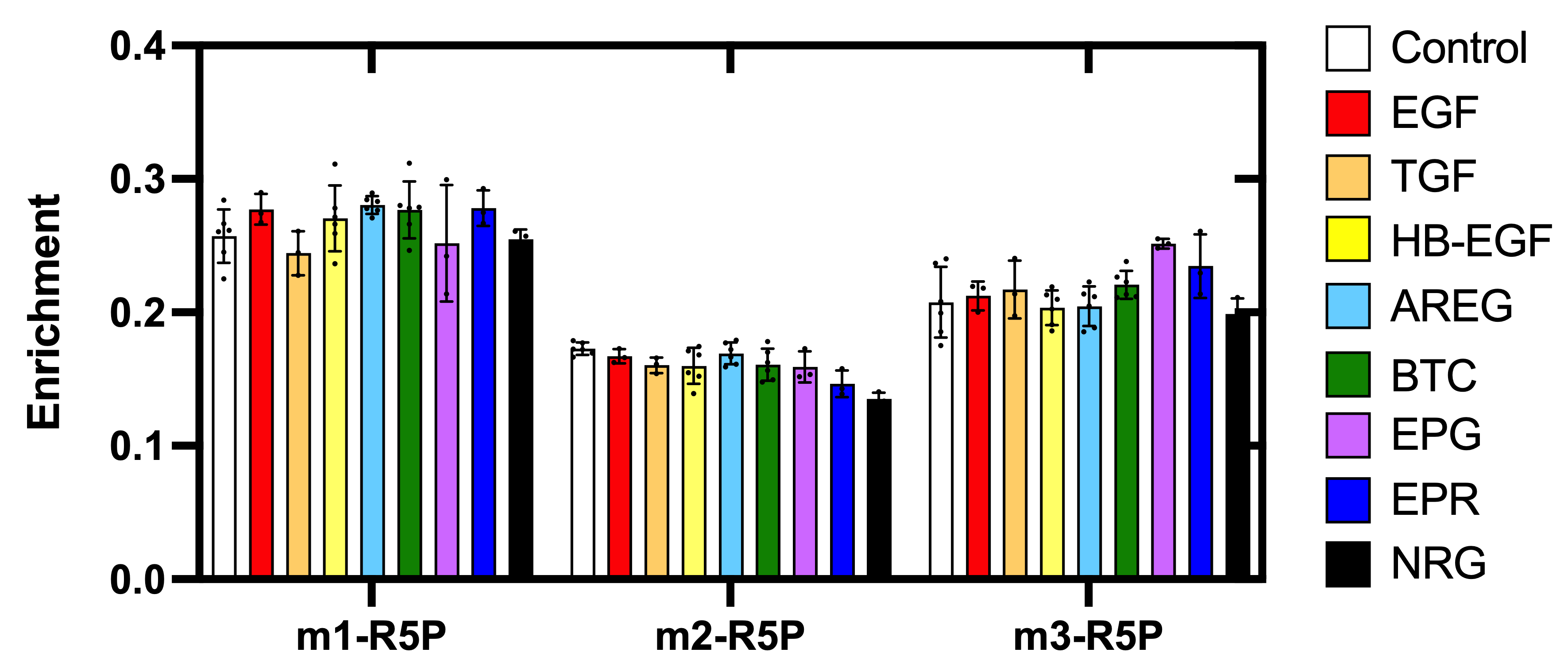


**Figure S4**. *Flux of [1,2-^13^C]-glucose into isotopologues of ribose-5P in MDA-MB-468 cells*. MDA-MB-468 cells were labeled with [1,2-^13^C] glucose, stimulated with ligand and processed for metabolomics analysis as outlined in Experimental Procedures. Bars represent the mean +/- standard deviation of triplicate or sextuplicate determinations.
